# Non-alcoholic fatty liver disease risk with GLP-1 receptor agonists and SGLT-2 inhibitors in type 2 diabetes: a nationwide nested case–control study

**DOI:** 10.1186/s12933-024-02461-2

**Published:** 2024-10-17

**Authors:** Kai-Cheng Chang, Fan-Chi Kuo, Chen-Yi Yang, Chun-Ting Yang, Huang-Tz Ou, Shihchen Kuo

**Affiliations:** 1 Department of Pharmacy, Linkou Chang Gung Memorial Hospital, Taoyuan, Taiwan; 2 School of Pharmacy, Institute of Clinical Pharmacy and Pharmaceutical Science, College of Medicine, National Cheng Kung University, 1 University Road, Tainan, 701 Taiwan; 3 Department of Pharmacy, National Cheng Kung University Hospital, Tainan, Taiwan; 4 School of Pharmacy, College of Medicine, National Cheng Kung University, Tainan, Taiwan; 5 Division of Metabolism, Endocrinology and Diabetes, Department of Internal Medicine, University of Michigan Medical School, Ann Arbor, MI USA

**Keywords:** Type 2 diabetes, Non-alcoholic fatty liver disease, Non-alcoholic steatohepatitis, Glucagon-like peptide-1 agonists, Sodium-glucose co-transporter-2 inhibitors

## Abstract

**Background:**

Non-alcoholic fatty liver diseases (NAFLDs)/non-alcoholic steatohepatitis (NASH) are the most common liver disorders among patients with type 2 diabetes. Newer classes of glucose-lowering agents (GLAs), such as glucagon-like peptide-1 receptor agonists (GLP-1RAs) and sodium-glucose cotransporter 2 inhibitors (SGLT2is), have been shown to improve liver-related biomarkers. However, their effects on the development of NAFLD/NASH remain inconclusive.

**Methods:**

A nested case–control study was conducted using Taiwan’s National Health Insurance Research Database for 2011–2018. Patients aged ≥ 40 years, diagnosed with type 2 diabetes, having stable non-insulin GLA use, and without NAFLD/NASH history were included. Patients with incident NAFLD/NASH were matched up to 10 randomly sampled controls based on individual’s age, gender, cohort entry date, type 2 diabetes diagnosis date, and disease risk score. Conditional logistic regression analyses were employed to estimate the association between liver risk and treatment exposure. Dose-response analysis was also performed.

**Results:**

There were 621,438 study patients included for analysis. During 1.8 years of median follow-up, the incidence of NAFLD/NASH was 2.7 per 1000 person-years. After matching, 5,730 incident NAFLD cases (mean age: 57.6 years, male: 53.2%) and 45,070 controls (57.7 years, 52.7%) were identified. Using GLP-1RAs or SGLT2is was associated with an insignificantly lower NAFLD/NASH risk (i.e., odds ratios [95% CIs]: 0.84 [0.46–1.52] and 0.85 [0.63–1.14], respectively) and increased cumulative SGLT2i doses were significantly associated with a reduced NAFLD/NASH risk (0.61 [0.38–0.97]).

**Conclusion:**

GLP-1RA and SGLT2i therapies in type 2 diabetes patients might prevent NAFLD/NASH development, with a significantly lower risk related to greater treatment exposure.

**Supplementary Information:**

The online version contains supplementary material available at 10.1186/s12933-024-02461-2.

## Background

Type 2 diabetes (T2D) shares several cardiometabolic and prognostic factors that are highly correlated with the development of non-alcoholic fatty liver disease (NAFLD) [1, 2]. About 60–80% of T2D patients have NAFLD or advanced liver diseases (i.e., non-alcoholic steatohepatitis [NASH], cirrhosis, hepatocellular carcinoma [HCC]) [3]. NAFLD will become the major etiology of HCC given the increasing coverage of the hepatitis B vaccine and rapid development of anti-viral drugs for hepatitis C virus (HCV) [4].

Although the U.S. Food and Drug Administration recently approved resmetirom for the treatment of adults with noncirrhotic NASH [5], lifestyle modification or alone, including smoking cessation, diet control, weight management, and exercise, remains essential [6–8]. For T2D patients with biopsy-proven NASH, thiazolidinediones (TZDs) might be a suitable treatment for steatohepatitis [9]. A systematic review of eight randomized trials showed that pioglitazone was associated with fibrosis improvement (odds ratio [OR]: 1.66, 95% confidence interval [CI]: 1.12–2.47) [10]. Recently, novel glucose-lowering agents (GLAs), namely glucagon-like peptide-1 receptor agonists (GLP-1RAs) [11–13] and sodium-glucose cotransporter 2 inhibitors (SGLT2is) [14, 15], were shown to improve liver fibrosis-related biomarkers such as alanine aminotransferase (ALT), visceral adipose tissue volume, and liver fat content. Unfortunately, a phase 2 study of semaglutide for NASH-related cirrhosis failed to achieve its primary goal (i.e., fibrosis improvement or NASH resolution) [16]; the pivotal phase 3 trial is still ongoing [17].

Despite the lack of trial evidence supporting the efficacy of GLP-1RAs or SGLT2is on the primary prevention of NAFLD, few real-world studies had evaluated the effects of GLP-1RAs and SGLT2is on incident NAFLD events [18–20]. Specifically, the UK Clinical Practice Research Datalink (CPRD) study found that use of SGLT2is was significantly associated with a lower risk of incident NAFLD events compared to that for use of dipeptidyl peptidase 4 inhibitors (DPP4is) [19]. An insignificantly reduced risk was also reported in a recent study of Korea populations [20]. Use of GLP-1RAs had an insignificantly reduced lower NAFLD risk compared to that for use of insulin [18] or DPP4i [19] based on the CPRD database. However, caution should be taken when interpreting these results due to short follow-up periods (i.e., 1.1–1.4 years [18–20]), the possibility of confounding bias caused by the inclusion of advanced T2D cases (i.e., requiring intensive insulin therapy [18]), uncertain liver effects (e.g., incident NAFLD) of active comparators (i.e., DPP4is [19, 20] and insulin [18]), lack of dose-response analysis, and limited generalizability to other ethnic populations.

The rapid increase of the prevalence of NAFLD among T2D patients has highlighted urgency for corroborating the hepatoprotective effects of newer GLA therapies. Therefore, we sought to investigate the risk of incident NAFLD associated with the use of GLP-1RAs and SGLT2is in a Chinese population with T2D.

## Methods

### Study design and database

We conducted a nested case–control study utilizing a nationwide cohort of patients with T2D derived from Taiwan’s National Health Insurance Research Database (NHIRD), which contains healthcare records for > 99% of Taiwan’s population of 23 million [21]. This database comprises comprehensive information at the individual level, including demographic data, diagnostic codes, procedure codes, and detailed prescriptions records. The nested case–control study design was chosen because it (1) enhances statistical efficiency (i.e., no need to classify treatment exposure for each person-moment of follow-up), (2) allows homogenous populations to be utilized, reducing the potential selection bias in conventional case–control study design, (3) minimize the concern of immortal time bias under a variety of cohort study designs [22–24], (4) accommodates rare events (i.e., incident NAFLD/NASH events according to previous UK and US studies), and (5) allows treatment dose–response analysis [25, 26]. This study was approved by the Institutional Review Board of National Cheng Kung University Hospital (A-EX-109-035).

### Cohort population

We included patients aged ≥ 40 years, diagnosed with T2D, and had newly initiated GLAs during the period of 2011 to 2018. The cohort entry date was defined as the first date of GLA prescription. We excluded patients without stable use of GLA (defined as having at least three refill records, with any gaps between two consecutive refills of no more than 90 days) and receiving any insulin therapies (considered as advanced T2D cases and thus at high risk for liver diseases). Patients with any prior NAFLD/NASH, cirrhosis, biliary cirrhosis, HCC, alcoholic liver disease, toxic liver diseases, hepatectomy, liver transplantation, autoimmune hepatitis, or inherited liver diseases were excluded to ensure a causal relationship between treatment exposure and liver outcomes in a time sequence. Women with gestational diabetes mellitus or polycystic ovarian syndrome were also excluded (Supplemental Fig. 1).

**Fig. 1:**
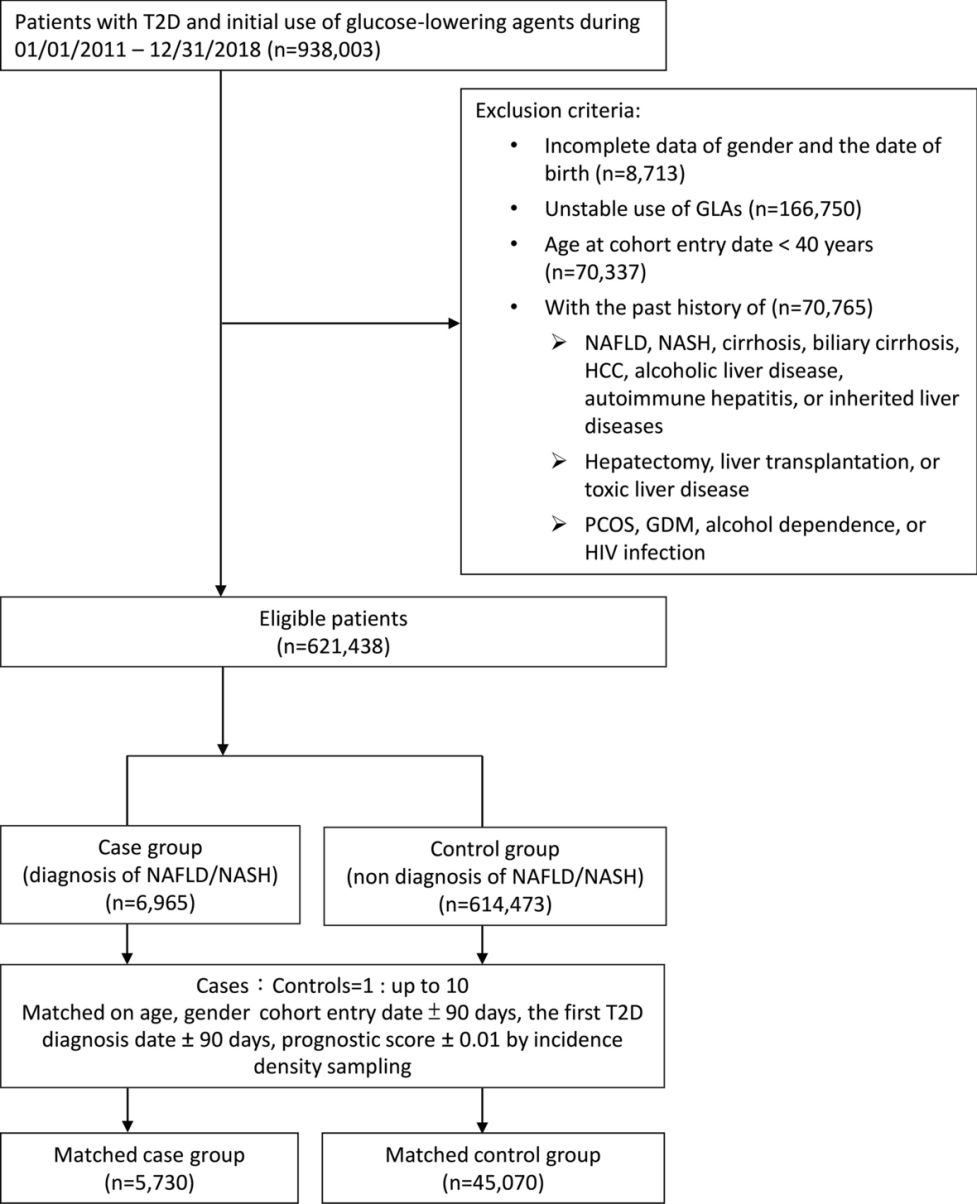
Cohort selection flow chart

### Definition of study outcomes

The main outcomes of interest were incident NAFLD/NASH events, which were ascertained when a patient had three consecutive outpatient diagnoses of NAFLD/NASH within 1 year or at least one inpatient diagnosis of NAFLD/NASH. We adopted a valid diagnosis coding system for NAFLD/NASH, namely code 571.8 in the International Classification of Diseases, Ninth Revision, Clinical Modification (ICD-9-CM), and code K75.81 in the ICD-10-CM, which had a positive predictive value of 92.0% in a previous study [27]. The first date of NAFLD/NASH diagnosis was defined as the index date. Of note, the term “NAFLD”, instead of “metabolic dysfunction-associated steatotic liver disease” (MASLD) defined by the latest American Association for the Study of Liver Diseases (AASLD) guidelines [28], was used in this study considering the high prevalence of hepatitis B virus (HBV) and HCV infections in Taiwan. [29, 30] Each study subject was followed from the cohort entry date (i.e., GLA initiation) until the occurrence of a study event of interest (i.e., incident NAFLD/NASH event), withdrawal from the National Health Insurance program, death, or the end of the study database (December 31, 2018), whichever came first.

### Case and control subject selection

Cases in the study cohort were defined as patients who experienced incident NAFLD/NASH events during follow-up after cohort entry (i.e., GLA initiation). For each case, up to 10 controls from risk set samples were randomly selected and matched on (1) age at the date of cohort entry, (2) gender, (3) cohort entry date (± 90 days), (4) T2D diagnosis date (± 90 days), and (5) disease risk score (DRS) (± 0.01), a summary score reflecting the severity of diabetes, and risk of occurrence of NAFLD/NASH and associated comorbidities before the cohort entry date, and measured using a logistic regression analysis [31]. The operational definition of NAFLD/NASH-associated risk factors is provided in Supplemental Table 1. The selection of case and control subjects is detailed in Supplemental Fig. 1.

**Table 1:** Risk of development of NAFLD events associated with use of individual glucose-lowering agents (GLAs) as compared to non-use

GLAs	No. (%) of cases (n = 5,730)		No. (%) of controls (n = 45,070)		Crude odds ratio (95% CI)		Adjusted odds ratio (95% CI)	
GLP-1 RAs as exposure (main analysis)								
No exposure	5,715	(99.74)	44,978	(99.80)	1	Ref.	1	
Ever use	15	(0.26)	92	(0.20)	1.11	(0.62–1.97)	0.84	(0.46–1.52)
Cumulative duration (days)^a^								
≤ 183	11	(0.19)	49	(0.11)	1.41	(0.70–2.84)	1.05	(0.51–2.17)
> 183	4	(0.07)	43	(0.10)	0.72	(0.25–2.07)	0.56	(0.19–1.64)
Cumulative dose (DDD)^a^								
≤ 180	10	(0.17)	47	(0.10)	1.35	(0.65–2.79)	1.04	(0.48–2.22)
> 180	5	(0.09)	45	(0.10)	0.83	(0.32–2.14)	0.62	(0.23–1.62)
SGLT-2 inhibitors as exposure (main analysis)								
No exposure	5,672	(98.99)	44,606	(98.97)	1	Ref.	1	Ref.
Ever use	58	(1.01)	464	(1.03)	0.99	(0.74–1.33)	0.85	(0.63–1.15)
Cumulative duration (days)^a^								
≤ 183	36	(0.63)	252	(0.56)	1.23	(0.85–1.78)	1.02	(0.70–1.49)
> 183	22	(0.38)	212	(0.47)	0.75	(0.48–1.19)	0.67	(0.42–1.06)
Cumulative dose (DDD)^a^								
≤ 143	36	(0.63)	232	(0.51)	1.37	(0.94–1.98)	1.13	(0.77–1.66)
> 143	22	(0.38)	232	(0.51)	0.68	(0.43–1.08)	0.61	(0.38–0.97)
Other regimen exposure (secondary analyses)								
TZDs	367	(6.40)	2,973	(6.60)	0.98	(0.87–1.10)	0.87	(0.77–0.98)
DPP-4 inhibitors	1,393	(24.31)	9,605	(21.31)	1.17	(1.10–1.26)	1.07	(1.00–1.15)
Incretin-based^b^	1,397	(24.38)	9,635	(21.38)	1.17	(1.09–1.26)	1.07	(1.00–1.15)
Weight-loss effect^c^	72	(1.26)	542	(1.20)	1.03	(0.79–1.35)	0.86	(0.65–1.12)
Newer GLAs^d^	1,418	(24.75)	9,836	(21.82)	1.17	(1.09–1.25)	1.06	(0.99–1.13)

DPP-4 inhibitors, dipeptidyl peptidase-4 inhibitors; GLP-1 RAs, glucagon-like peptide-1 receptor agonists; SGLT-2 inhibitors, sodium-glucose cotransporter-2 inhibitors; GLAs, glucose-lowering agents; TZDs, thiazolidinediones; DDD, defined daily dose

^a^The cut-off values for cumulative duration and dose were determined based on the median values

^b^Incretin-based agents included DPP-4 inhibitors and GLP-1 RAs

^c^GLP-1 RAs and SGLT-2 inhibitors had a weight-loss effect

^d^Newer GLAs included DPP-4 inhibitors, GLP-1 RAs, and SGLT-2 inhibitors

### Exposure measurement

Use of GLA were measured according to the World Health Organization Anatomical Therapeutic Chemical Classification System (Supplemental Table 2). The primary exposure was the ever use of GLP-1RAs or SGLT2is, defined as the presence of at least one prescription between the date of cohort entry and the year before the index date. Among ever users, we further defined long-term users as those who had a cumulative defined daily dose or a duration of GLA use higher than the median values. Additional analyses categorized the exposure of GLAs into incretin-based agents (i.e., DPP4is and GLP-1RAs), weight-loss effect agents (i.e., GLP-1RAs and SGLT2is), and newer GLAs (i.e., DPP4is, GLP-1RAs, and SGLT2is) (Supplemental Table 2). Patients without exposure to GLP-1RAs or SGLT2is were placed into the reference group for the analyses.

### Measurement of covariates

Patient clinical characteristics and associated prescriptions were measured during the year before the cohort entry date. They included diabetes-related complications (i.e., nephropathy, neuropathy, retinopathy, peripheral vascular diseases, hypertension, myocardial infarction, cardiac arrhythmia, heart failure, and stroke), NAFLD/NASH-associated comorbidities (i.e., chronic hepatitis B virus [HBV], HCV infection, HBV carrier, obesity, dyslipidemia, hypothyroidism, obstructive sleep apnea, psoriasis, and gout), and drugs (e.g., amiodarone, methotrexate, systemic corticosteroids, valproate, carbamazepine, tamoxifen, tocopherol, and antiviral agents) that may be associated with increased risk of steatohepatitis. To minimize potential unmeasured confounding caused by a lack of data regarding patients’ body mass index (BMI), glycated hemoglobin (HbA1c), lipid profile, and frailty, we also measured the receipt of bariatric surgery (as a proxy for a person having BMI ≥ 35 kg/m^2^ in a Taiwanese setting), diabetes complications severity index (DCSI), numbers of HbA1c and low-density lipoprotein (LDL) tests, and hospital admissions as surrogate indicators. The above variables are detailed in Supplemental Table 1. To address potential time-dependent bias, we also measured the above variables in the year before the index date, except for primary exposures (i.e., GLP-1RAs and SGLT2is), to estimate the DRS using a logistic regression analysis [31] and adjusted the score in the analyses (i.e., NAFLD/NASH events) (Supplemental Fig. 1).

### Statistical analysis

The crude incidence rate of NAFLD events was estimated as the number of events per 1,000 person-years. The difference between cases and the control in patient characteristics was tested using the standardized mean difference (SMD), with a value greater than 0.1 indicating a statistically significant difference. Multivariable conditional logistic regression was used to estimate ORs and associated 95% CIs of events with treatment exposure. All analyses were performed using the statistical software SAS 9.4 (SAS Institute Inc.).

### Sensitivity and subgroup analyses

We conducted several sensitivity and subgroup analyses to test the robustness of our findings. First, to minimize misclassification bias, ever users were re-defined and restricted to patients with stable use of GLAs (i.e., GLP-1RA or SGLT2i) in the analyses (defined as at least three GLA prescriptions with any gaps between two consecutive refills of less than 30 days). Second, we excluded patients who had any TZD records during follow-up from the cohort entry date (i.e., GLA initiation) to the index date (i.e., liver event occurrence), given its potential liver benefit. Third, we used a lag-time approach to address protopathic bias. [32, 33] That is, we excluded patients who had NAFLD/NASH events within 30, 60, 90, 180, and 365 days following cohort entry because these subjects were considered as high-risk patients for liver events. Fourth, instead of total follow-up for treatment exposure in the primary analyses, we only assessed the exposure of GLAs in the 2 years prior to the index date (i.e., liver event occurrence) to strength the relationship between the study event and exposure. Fifth, we used a positive control exposure to rule out potential confounding bias, thereby validating our study protocol and affirming database quality. That is, we estimated the risk of NAFLD/NASH with TZD use given the compelling liver benefit of TZD therapy [9]. Sixth, we also applied an array approach to estimate the impact of unmeasured confounding (i.e., BMI) [34]. Lastly, we conducted a series of subgroup analyses stratified by age (< 65 and ≥ 65 years) at cohort entry, gender, and the presence of dyslipidemia in the year before/at the cohort entry date.

## Results

**Fig. 2:**
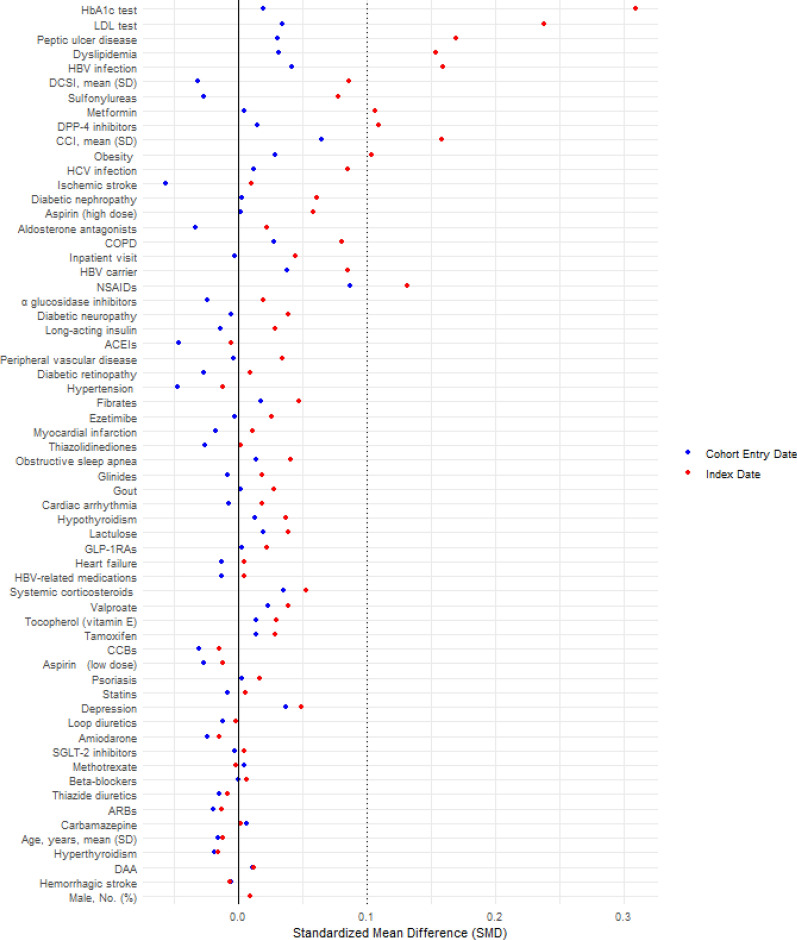
Absolute standardized mean differences of baseline characteristics between cases and controls at cohort entry date and index date

Of the 621,438 T2D patients newly treated by GLAs between 2007 and 2018, we included 5,730 cases and their matched controls (45,070 patients). With a mean follow-up of 1.8 years, the incidence rate of NAFLD/NASH was 2.78 per 1,000 person-years. The study flow chart is detailed in Fig. 1. Supplemental Table 3 shows the characteristics at cohort entry and the index date for the case and control groups. All patient characteristics at cohort entry between the cases and controls were comparable (Fig. 2). Several variables related to the occurrence of NAFLD during follow-up were significantly different between the cases and controls, including HBV infection, dyslipidemia, obesity, peptic ulcer disease, HbA1c test number, LDL test number, and the use of metformin, DPP4is, and NSAIDs. Therefore, we estimated the DRS based on these variables and further adjusted the score using multivariable conditional logistic regression analyses.

Use of GLP-1RAs was associated with an insignificantly lower risk of NAFLD/NASH events (adjusted OR [aOR]: 0.84 [95% CI: 0.46–1.52]). A longer duration (> 183 days) and a higher cumulative dose (> 180 defined daily dose) of GLP-1RA therapy were associated with an insignificantly reduced risk of NAFLD/NASH events (i.e., 0.56 [0.19–1.64] and 0.62 [0.23–1.62], respectively; Table 1).

Similarly, SGLT2i users had an insignificantly lower risk of NAFLD/NASH events (aOR: 0.85 [95 CI%: 0.63–1.15]) compared to non-users. A statistically significantly lower risk among those with a cumulative SGLT-2i dose of more than 143 defined daily dose (0.61 [0.38–0.97]) was observed. Furthermore, TZD use, as a positive control exposure in our study, was associated with a lower risk of NAFLD/NASH events (aOR: 0.87 [95% CI: 0.77–0.98]). Similar findings were observed in GLA regimens with a weight-loss effect (i.e., GLP-1RAs and SGLT2is) (i.e., 0.86 [0.65–1.12]; Table 1).

**Fig. 3:**
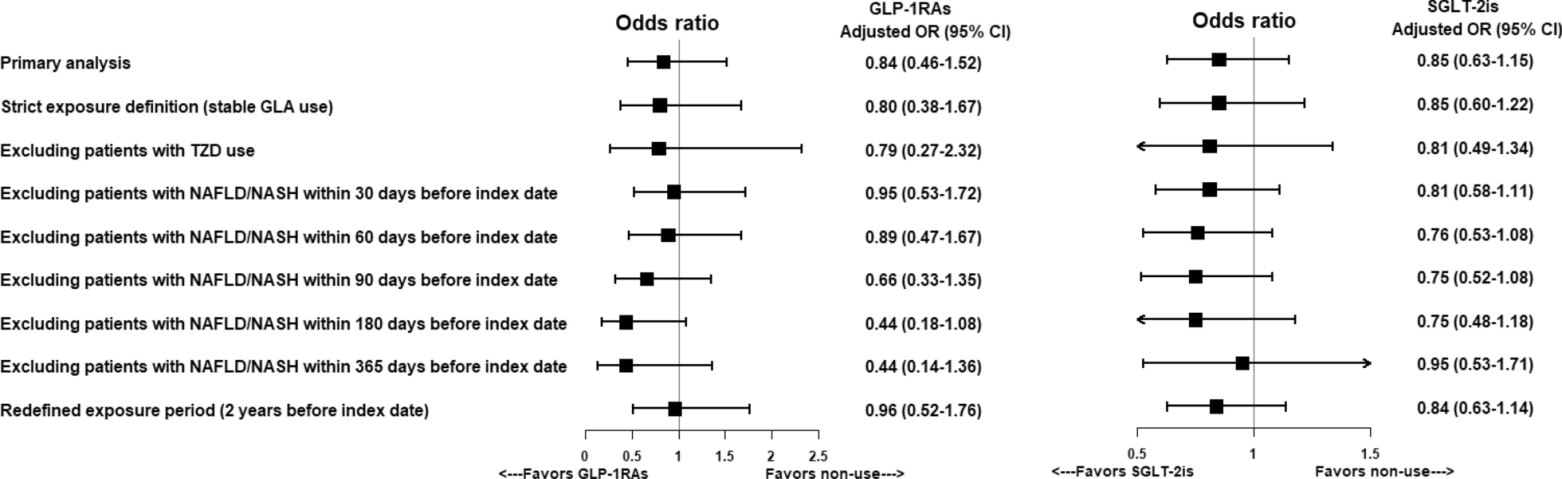
Risk of development of NAFLD events associated with use of GLP-1RAs or SGLT2is as compared to non-use (primary and sensitivity analyses)

**Fig. 4:**
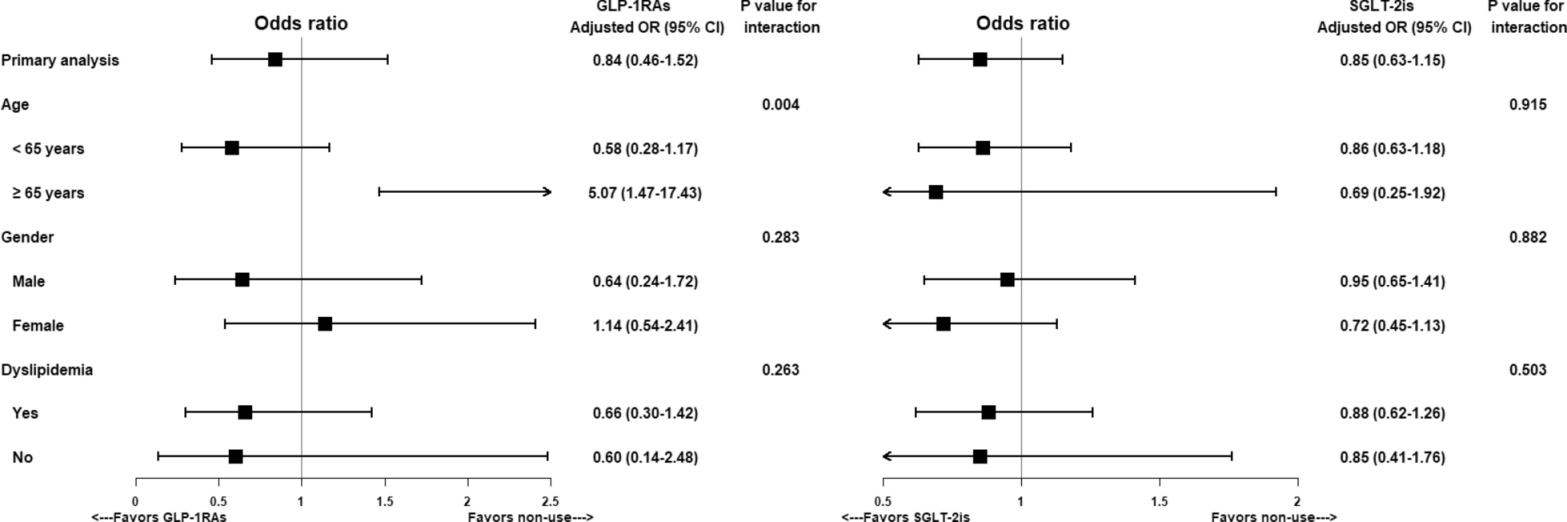
Risk of development of NAFLD associated with use of GLP-1RAs or SGLT2is as compared to non-use (primary and subgroup analyses)

All sensitivity analyses (i.e., using a strict definition [stable use] for previous or current users of GLP-1RAs or SGLT2is, excluding the TZD effect, using the lag-time approach, and refining the treatment exposure period as 2 years before the index date) yielded results that are consistent with the primary findings, showing a decreased but insignificant risk of NAFLD/NASH events with the use of GLP-1RAs or SGLT-2is (Fig. 3). Moreover, in the array approach, we considered BMI as a strong unmeasured confounder in our study and assumed a different prevalence of BMI ≥ 27 kg/m^2^ among GLP-1RA users and non-users. The results show that the effect of GLP-1RAs on NAFLD/NASH becomes statistically significant when BMI is adjusted for (Supplemental Fig. 2). The results of subgroup analyses are consistent with our main results, except for patients aged ≥ 65 years, who had an increased risk of NAFLD/NASH associated with GLP-1RA use versus non-use (aOR: 5.07 [95% CI: 1.47–17.43]) (Fig. 4).

## Discussion

This study is the first to assess the effect of newer GLAs (i.e., GLP-1RAs and SGLT2is) on the development of NAFLD/NASH in an ethnic Chinese population. By utilizing 8 years of longitudinal data from a nationwide population with T2D, we captured more NAFLD/NASH events (i.e., 5,730) that developed in the study period compared to those reported in previous studies (i.e., 1,998–2,526 [18–20]). A trend of a lower risk of incident NAFLD/NASH events associated with GLP-RA and SGLT2i use was observed, with a significantly reduced risk related to increased exposure to SGLT2i treatment. Hence, these GLAs, which have a weight-loss effect, might alleviate the risk of developing NAFLD/NASH for patients with T2D. Consistent results across several sensitivity and subgroup analyses not only support the validity of our findings but also enhance their applicability to real-world patients with T2D.

### SGLT2is and NAFLD/NASH risk

Although the exact mechanisms of the hepatic effect of SGLT2i therapy remain unclear, several possible hypotheses have been proposed to support the decreased risk of NAFLD following this treatment. First, SGLT2is have a weight-loss effect, which is essential for the management of NALFD/NASH. Second, SGLT2is have pleiotropic effects on metabolic diseases, including anti-inflammation [35], blood pressure reduction, lipid profile improvement, and a lowering of the risk of cardiovascular diseases and chronic kidney diseases, of which NAFLD is considered to be the hepatic manifestation. Beyond the biological evidence, existing systematic reviews suggest the potential hepatic benefits of SGLT2i therapy for T2D patients [36–38]. That is, compared to placebo (non-use) or active comparators (i.e., metformin, TZD, and insulin), use of SGLT2is significantly decreased visceral adipose tissue volume and liver enzymes (i.e., alanine aminotransferase [ALT], aspartate aminotransferase [AST]). Of note, this review evidence [36–38] only included patients with established NAFLD/NASH. Only two previous observational studies, one from UK [19] and one from Korea [20], investigated the effect of SGLT2i therapy for preventing the development of NAFLD/NASH in T2D patients. They showed marginally significantly reduced risks of incident NAFLD/NASH events following use of SGLT2is (i.e., aORs [95% CIs]: 0.79 [0.64–0.96] [19] and 0.93 [0.80–1.08] [20], respectively). However, caution should be taken when interpreting these results because the primary analyses in these studies [19, 20] were based on the as-treated scenario, which may limit the applicability of these results to patients with optimal treatment adherence. In addition, these analyses are prone to informative censoring (when adherence was estimated on censored data), thereby causing potential bias in the efficacy analysis and masking the true treatment differences in real-world settings, where suboptimal adherence or treatment discontinuation is common.

In contrast, our primary analysis was based on the intention-to-treat scenario, which acknowledges the complexity of drug therapies in managing T2D and thus does not censor (stop the observation of) a patient when the treatment pattern changes, thereby enhancing the applicability of our results to real-world populations. In addition, our analysis findings revealed high persistence rates in the use of SGLT2is [medication possession ratio (MPR): 93.1%] and GLP-1RAs (MPR: 88.8%) among our study patients, thereby minimizing the potential of misclassification bias caused by the intention-to-treat scenario in a study with a long-term observational period. Similarly, our study found that use of SGLT2is was associated with a lower but insignificant risk of incident NAFLD/NASH events (i.e., aOR [95% CI]: 0.85 [0.63–1.15]). In addition, patients with a higher cumulative dose of SGLT2i had a significantly decreased incidence of NAFLD/NASH (i.e., aOR [95% CI]: 0.61 [0.38–0.97]). This result is supported by a previous animal study that showed a reduction in liver enzymes and insulin resistance associated with increased dapagliflozin dose [39]. Hence, a longer exposure or higher dose of SGLT2i therapy is suggested for T2D patients at high risk for the development of NAFLD/NASH.

### GLP-1RAs and NAFLD/NASH risk

The potential mechanisms of GLP-1RAs for preventing NAFLD/NASH events are similar to those of SGLT2is (i.e., weight-loss and metabolic effects). Previous studies investigated the effect of current GLP-1RA use on incident NAFLD/NASH events in relatively short follow-ups (i.e., 26 weeks [18] and 1.4 year [19]) and found that the results depended on the active comparator [18, 19]. Specifically, GLP-1RA users were associated with a higher risk of the development of NAFLD/NASH compared to current insulin users (aHR [95% CI]: 1.22 [0.91–1.63]) [18] but a decreased risk compared to current users of sulfonylureas (0.85 [0.64–1.13]) [18] or DPP4i (0.83 [0.66–1.63]) [19]. Of note, insulin users, as active comparators, represented more advanced T2D patients (i.e., requiring intensive glycemic control), and thus confounding by indication in these selective patients cannot be ruled out; such confounding could mask the beneficial liver effect of GLP-1RAs [18]. For example, for patients with obesity or high BMI levels, physicians might not prescribe insulin considering its potential weight gain effect.

The present study also found that GLP-1RA use reduced the risk of NAFLD/NASH despite insignificant results (i.e., aOR [95% CI]: 0.84 [0.46–1.52]). In addition, our dose–response results were consistent with the findings of a phase 2 trial of semaglutide among patients with NASH [40], showing dose-dependent reductions in liver-related biomarkers (i.e., ALT, AST, and cytokeratin-18 fragments). Hence, GLP-1RAs may also be recommended to patients with T2D, especially those with obesity, at the maximum tolerated dose for its adverse events (e.g., gastrointestinal side effects) as a preventive strategy against NAFLD/NASH.

### Potential limitations

First, the low number of SGLT2i users (given its listing in Taiwan’s National Health Insurance program since 2016) or GLP-1RA users (due to restricted reimbursement criteria by Taiwan’s National Health Insurance program) in our study period (i.e., 2007–2018) may affect the study’s power to detect statistical significance. Second, this study of a Taiwanese population might only be generalizable to ethnic Chinese populations. Third, while a nested case–control design is effective for evaluating associations, it may have a limited ability to infer causality compared with a cohort design. This is particularly true when assessing drug effects, where cohort studies with active comparators can offer more robust causal insights. So, future studies using a cohort design with active comparators, such as metformin or DPP4i, would help validate our findings. Fourth, potential unmeasured confounding effects (e.g., HbA1c and BMI) on the study results may exist. However, we mitigated potential unmeasured confounding effects by employing proxy variables, (e.g., DCSI was used for diabetes severity and obesity surgery was used to identify morbidly obese patients). In addition, we implemented a rule-in approach (i.e., array approach) to estimate the impact of BMI on GLP-1RA effectiveness. The results indicate that when BMI is adjusted for in the analyses, GLP-1RAs exhibit greater efficacy in preventing NAFLD/NASH. Further large-scale multi-country studies based on the target trial emulation approach, which can eliminate the biases and confounding effects commonly seen in observational studies, using real-world data are warranted to confirm our findings. Fifth, the recent AASLD guidelines redefined the diagnostic criteria of metabolic liver diseases, namely MASLD [28]. Most of our study patients would meet the criteria for MASLD, since they were T2D patients experiencing steatotic liver disease events. However, approximately 5% of study patients also had co-existing viral hepatitis (i.e., HBV and HCV), which could not be fully excluded in our study setting (i.e., Taiwan [29, 30]). Future studies based on the MASLD definition for study outcomes remain warranted to corroborate our findings.

## Conclusions

Use of SGLT2is or GLP-RAs might be associated with decreased risks of incident NAFLD/NASH events, especially longer exposure or higher dose of SGLT-2i therapy. These treatments should thus be prioritized for patients with T2D at risk for the development of NAFLD/NASH (e.g., obesity).

## Supplementary Information

Supplementary Material 1.

## Data Availability

Data sharing is not applicable to this study as data management and analysis were only allowed to be conducted in Health and Welfare Data Science Center in Taiwan for data privacy and safety concerns.

## Abbreviations

T2D: Type 2 diabetes
NAFLD: Non-alcoholic fatty liver disease
NASH: Non-alcoholic steatohepatitis
HCC: Hepatocellular carcinoma
TZD: Thiazolidinediones
GLAs: Glucose-lowering agents
GLP-1RAs: Glucagon-like peptide-1 receptor agonists
SGLT2i: Sodium-glucose cotransporter 2 inhibitors
ALT: Alanine aminotransferase
DPP4is: Dipeptidyl peptidase 4 inhibitors
DRS: Disease risk score
HBV: Hepatitis B virus
HCV: Hepatitis C virus
BMI: Body mass index
HbA1c: Glycated hemoglobin
DCSI: Diabetes complications severity index
LDL: Low-density lipoprotein
SMD: Standardized mean difference
