# Supplementary material for: Non-alcoholic fatty liver disease risk with GLP-1 receptor agonists and SGLT-2 inhibitors in type 2 diabetes: a nationwide nested case–control study
Source: Cardiovasc Diabetol. 2024 Oct 17;23:367. doi: 10.1186/s12933-024-02461-2 (PMC11487834; doi:10.1186/s12933-024-02461-2)
Supplement: Supplementary file 1 — Supplementary Material 1. [file 12933_2024_2461_MOESM1_ESM.docx]

**Supplementary data**

**Supplemental Figure 1.** Overview of Nested Case-Control Design

**Supplemental Table 1.** Diagnosis Codes Used to Define Exclusion Criteria and NAFLD-Related Risk Factors

**Supplemental Table 2.** WHO ATC Codes Used to Define Class of Glucose-Lowering Agents and Comedications

**Supplemental Table 3.** Baseline Characteristics of Patients in Case and Control Groups Measured from Year Before/at Cohort Entry Date and Index Date

**Supplemental Figure 2.** Impact of Unmeasured Confounders Assessed Using Rule-Out Method


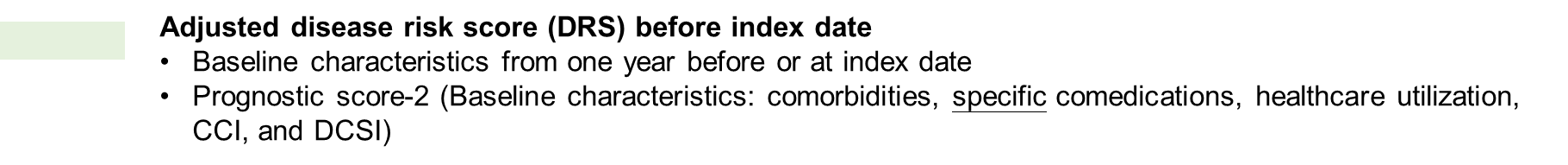

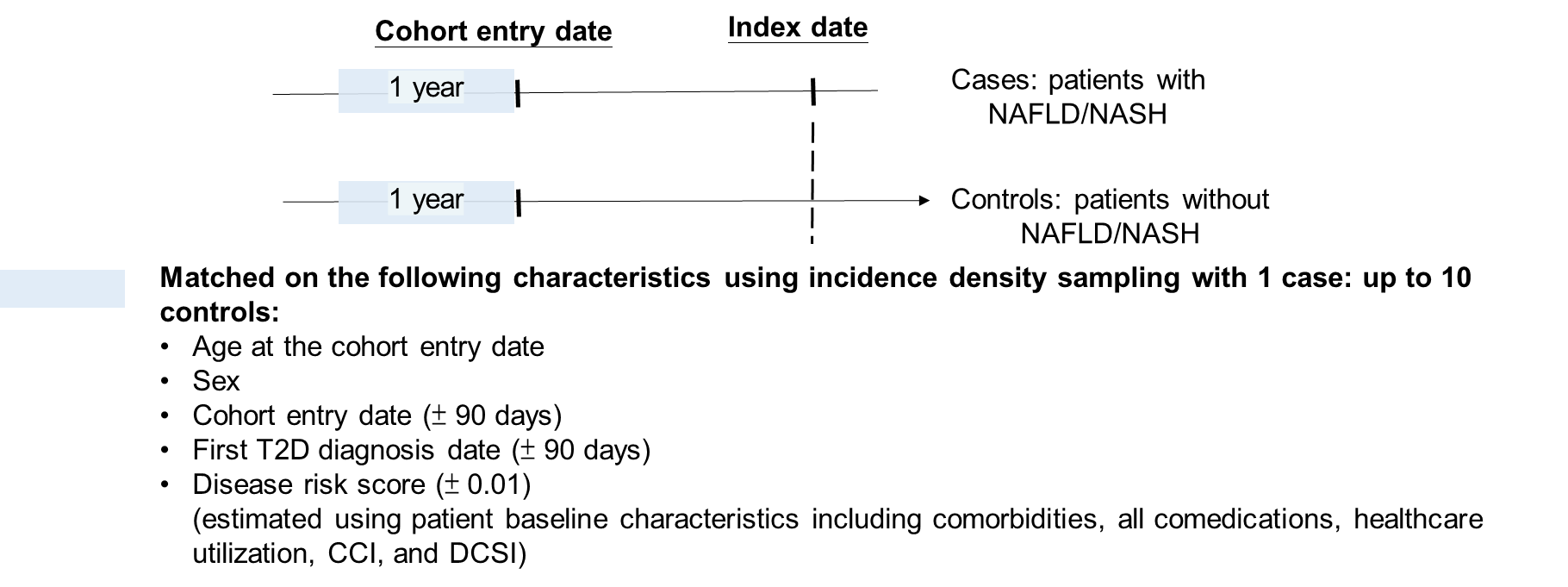

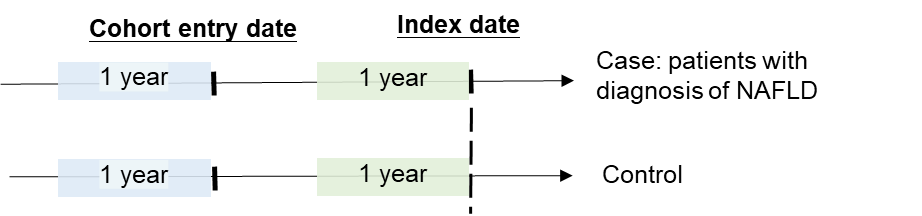

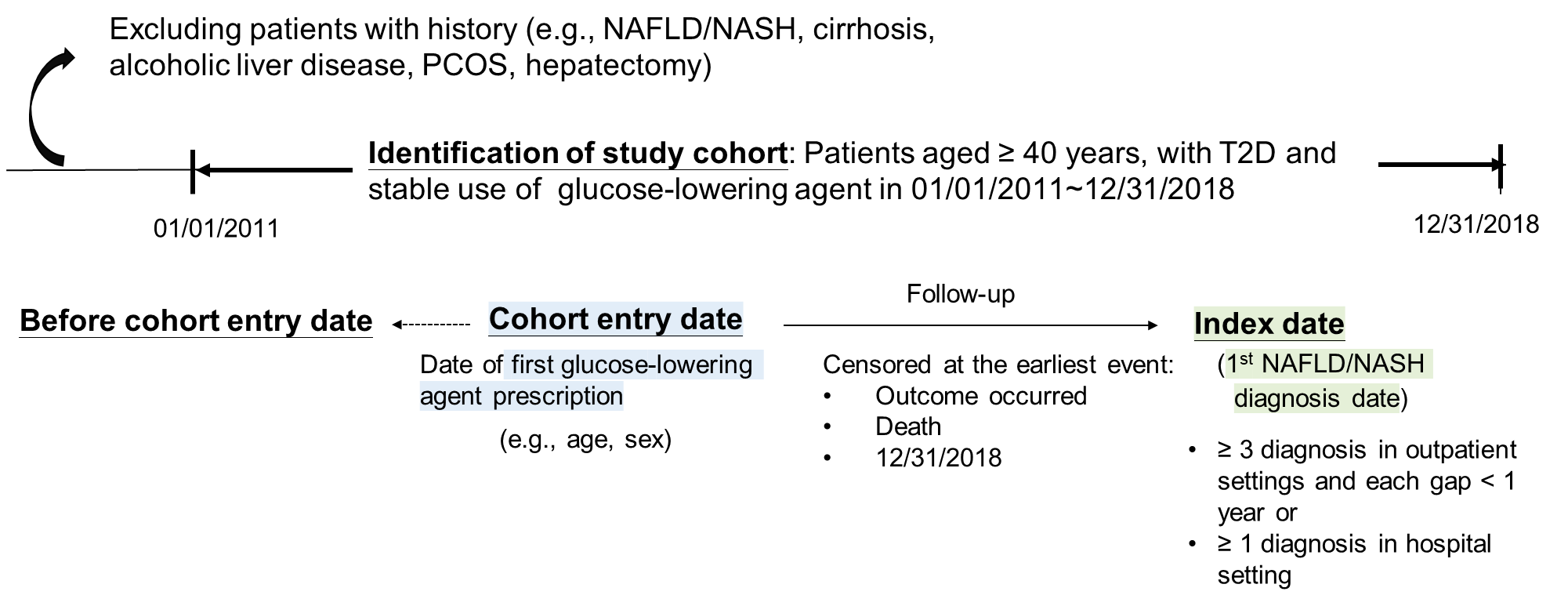
**Supplemental Figure 1. Overview for nested case-control design**

Abbreviations: T2D, type 2 diabetes; DCSI, Diabetes Complications Severity Index; CCI, Charlson Comorbidity Index.

**Supplemental Table 1. Diagnosis codes used to define exclusion criteria and NAFLD-related the risk factors**

| Definition variable | ICD-9-CM & ICD-10-CM codes | Taiwan National Health Insurance code |
| --- | --- | --- |
| **Exclusion criteria** | |  |
| Hepatocellular carcinoma (HCC) | 155.0, C22.0, C22.9 | - |
| Non-alcoholic fatty liver disease (NAFLD) | 571.8, K75.81 | - |
| Cirrhosis | 456.0, 456.2, 571.5, 572.2, 572.4, 789.5, I85.0, I85.1, K72.90, K72.91, R18.8, K74.0−K74.2, K74.60, K74.69 | - |
| Biliary cirrhosis | 571.6, K74.3−K74.5 | - |
| Alcoholic liver disease  and alcohol dependence | 571.0−571.3, 303.9, V11.3, V79.1, K70 | - |
| Polycystic ovary syndrome (PCOS) | 256.4, E28.2 | - |
| Gestational diabetes | 648.0, O24 | - |
| Toxic liver disease | 573.3, K71 | - |
| Other infection cause hepatitis | 573.2, K77 | - |
| Autoimmune hepatitis | 571.42, K75.4 | - |
| Human immunodeficiency virus (HIV) infection | 042, V08, 079.53, B20, Z21, Z22.6, B97.35 | - |
| Hemochromatosis | 275, E83.11 | - |
| Wilson’s disease | 275.1, E83.01 | - |
| α1-antitrypsin deficiency | 277.6, E88.01 | - |
| Budd-Chiari syndrome | 453.0, I82.0 | - |
| Hepatectomy  or liver transplantation |  | 75002B, 75003B, 75004B, 75005B, 75020A, 75020B, 75015B, 75016B, 75017B, 75018B, N26021, N26018, N26019, 75025B, 75026B, 75027B, 75028B, 75030B, 75031B, 75032B, 75033B |
| **Risk factors** | |  |
| Diabetic nephropathy | 250.4, 249.4, 580, 581, 582, 583, 585, 586, 593.9, E08.3, E11.2, E13.2, N00, N04, N03, N05, N18, N19 | - |
| Diabetic neuropathy | 250.6, 249.6, 337.0, 337.1, 354, 355, 356.9, 357.2, 358.1, 458.0, 536.3, 564.5, 596.54, 713.5, 951.0, 951.1, 951.3, E08.4, E11.4, E13.4, G90.09, G90.8, G90.9, G99.0, G56, G57, G60.9, G73.3, G90.01, H49, I95.1, K31.84, K59.1, N31.9, M14.6, S04 | - |
| Peripheral vascular disease | 250.7, 249.7, 442.3, 440.21, 443.81, 443.9, 892.1, 040.0, 444.22, 707.1, 785.4, E08.5, E11.5, E13.5, I96, E08.621, E11.621, E13.621, I72.4, I70.21, I73.89, I73.9, S91.3, A48.0, I74.3, L97 | - |
| Diabetic retinopathy | 250.5, 361, 362, 369, 379.23, E08.3, E11.3, E13.3, H35.0, H35.35, H35.6, H35.8, H35.9, H33, H54, H43.1 | - |
| Hepatitis B virus (HBV) infection | 070.22, 070.23, 070.32, 070.33, 070.20, 070.30, B18.0, B18.1, B19.10, B19.11 | - |
| Hepatitis C virus (HCV) infection | 070.41, 070.51, 070.44, 070.54, B18.2, B19.20, B19.21 | - |
| HBV carrier | V02.61, B17.0, Z22.51 | - |
| Dyslipidemia | 272.0−272.5, 272.8−272.9,  E78.0−E78.6, E78.70, E78.79, E78.81, E78.89, E78.9 | - |
| Hypothyroidism | 243, 244, E00, E01.8, E02, E03.0, E03.1, E03.2, E03.3, E03.8, E03.9, E89.0 | - |
| Obstructive sleep apnea | 780.51, 780.53, 780.57, G47.33 | - |
| Psoriasis | 696.0, 696.1, L40 | - |
| Gout | 274, M10.0 | - |
| Obesity | 278.00–278.02, 278.1, E65, E66.01, E66.09, E66.1, E66.2, E66.8, E66.9 | - |
| Hypertension | 401–405, I10, I11, I12, I13, I15, I16 | - |
| Myocardial infarction | 410, 411, 412, I21, I22, I23, I24, I25.2 | - |
| Cardiac arrhythmia | 426, 427, I44, I45, I47-I49 | - |
| Heart failure | 428, I50 | - |
| Ischemic stroke | 433, 434, 437.1, I63, I65, I66, I67.81, I67.82, I67.89 | - |
| Hemorrhagic stroke | 430, 431, 432, I60, I61, I62 | - |
| Chronic obstructive pulmonary disease (COPD) | 491, 492, 496, J41, J42, J43, J44 | - |
| Depression | 296.2, 300.4, 311, F32, F34.1 | - |
| Peptic ulcer disease | 533, K27 | - |
| Hyperthyroidism | 242, E05 | - |
| HbA1c tests |  | 09006B, 09006C |
| LDL tests |  | 09044C |

**Supplemental Table 2. WHO ATC code used to define the class of glucose-lowering agents and comedications**

| Definition drugs | WHO ATC code |
| --- | --- |
| Amiodarone | C01BD01 |
| Methotrexate | L01BA01 |
| Systemic corticosteroids | H02 |
| Valproate | N03AG01 |
| Carbamazepine | N03AF01 |
| Tamoxifen | L02BA01 |
| Metformin | A10BA02, A10BD02, A10BD03, 10BD05, A10BD07, A10BD08, 10BD10, A10BD11, A10BD13, 10BD14, A10BD15, A10BD20 |
| Sulfonylureas | A10BB, A10BD02 |
| Thiazolidinediones | A10BG, A10BD03, A10BD05, A10BD09 |
| Dipeptidyl peptidase-4 (DPP-4) inhibitors | A10BH, A10BD07, A10BD08, A10BD09, A10BD10, A10BD11, A10BD13, A10BD19, A10BD21, A10BD24 |
| Glucagon-like peptide-1 receptor agonists (GLP-1RAs) | A10BJ, A10AE54, A10AE56 |
| sodium-glucose cotransporter 2 (SGLT-2) inhibitors | A10BK, A10BD15, A10BD19, A10BD20, A10BD21, A10BD24 |
| α glucosidase inhibitors | A10BF |
| Glinides | A10BX02, A10BX03, A10BX08,  A10BD14 |
| Long acting insulin | A10AD, A10AE |
| Statins | C10AA, C10BA01, C10BA02, C10BA03, C10BA05, C10BX03 |
| Fibrates | C10AB, C10BA03 |
| Ezetimibe | C10AX09, C10BA02, C10BA05 |
| Angiotensin-converting enzyme inhibitors (ACEIs) | C09AA, C09BB, C09BA |
| Angiotensin receptor blockers (ARBs) | C09CA, C09DB, C09DA, C09DX |
| Beta blockers | C07AA, C07AB, C07AG |
| Calcium Channel Blockers (CCBs) | C08D, C08CA, C09BB, C09DB~~,~~ C09DX01, C09DX03, C10BX03 |
| Loop diuretics | C03BA, C03CA, C09BA04, C09DA09 |
| Thiazide diuretics | C03AA, C09BA01, C09BA02, C09DA01, C09DA03, C09DA04, C09DA06, C09DA07, C09DA08, C09DX01, C09DX03 |
| Aldosterone antagonists | C03DA |
| Aspirin (low dose) | B01AC06 |
| Aspirin (high dose) | N02BA01 |
| non-steroidal anti-inflammatory drugs (NSAIDs) | M01A |
| Lactulose | A06AD11 |
| Tocopherol (vitamin E) | A11HA03 |
| Direct antivirals agents (DAAs) | J05AP |
| Antivirals for treatment of HBV infections | J05AF05, J05AF08, J05AF10, J05AF11, J05AF13, J05AF07 |

**Supplemental Table 3. Baseline Characteristics of Patients in Case and Control Groups Measured from Year Before/at Cohort Entry Date and Index Date**

|  | **At cohort entry date** | | | | | **At index date^a^** | | | | |
| --- | --- | --- | --- | --- | --- | --- | --- | --- | --- | --- |
| **Characteristics** | **Cases**  **(n=5,730)** | | **Controls**  **(n=45,070)** | | **SMD^b^** | **Cases**  **(n=5,730)** | | **Controls**  **(n=45,070)** | | **SMD^b^** |
| Age, years, mean (SD) | 57.60 | (10.46) | 57.77 | (10.44) | -0.016 | 59.28 | (10.58) | 59.41 | (10.57) | -0.012 |
| Male, No. (%) | 3,047 | (53.18) | 23,753 | (52.70) | 0.009 | 3,047 | (53.18) | 23,753 | (52.70) | 0.009 |
| **Diabetes-related comorbidities, No. (%)** | | | | | | | | | | |
| Diabetic nephropathy | 414 | (7.23) | 3,225 | (7.16) | 0.003 | 909 | (15.86) | 6,168 | (13.69) | 0.061 |
| Diabetic neuropathy | 322 | (5.62) | 2,587 | (5.74) | -0.005 | 499 | (8.71) | 3,449 | (7.65) | 0.039 |
| Peripheral vascular disease | 169 | (2.95) | 1,360 | (3.02) | -0.004 | 250 | (4.36) | 1,667 | (3.70) | 0.034 |
| Diabetic retinopathy | 210 | (3.66) | 1,884 | (4.18) | -0.027 | 479 | (8.36) | 3,659 | (8.12) | 0.009 |
| **Liver-related diseases, No. (%)** | | | | | | | | | | |
| HBV infection | 258 | (4.50) | 1,657 | (3.68) | 0.042 | 422 | (7.36) | 1,682 | (3.73) | **0.159** |
| HCV infection | 98 | (1.71) | 700 | (1.55) | 0.012 | 181 | (3.16) | 824 | (1.83) | 0.085 |
| HBV carrier | 40 | (0.70) | 188 | (0.42) | 0.038 | 69 | (1.20) | 198 | (0.44) | 0.085 |
| **Other risk factors associated with NAFLD, No. (%)** | | | | | | | | | | |
| Dyslipidemia | 2,960 | (51.66) | 22,568 | (50.07) | 0.032 | 3,744 | (65.34) | 26,076 | (57.86) | **0.154** |
| Hypertension | 3,090 | (53.93) | 25,356 | (56.26) | -0.047 | 3,355 | (58.55) | 26,647 | (59.12) | -0.012 |
| Hypothyroidism | 60 | (1.05) | 413 | (0.92) | 0.013 | 78 | (1.36) | 436 | (0.97) | 0.037 |
| Obstructive sleep apnea | 53 | (0.92) | 359 | (0.80) | 0.014 | 59 | (1.03) | 294 | (0.65) | 0.041 |
| Psoriasis | 37 | (0.65) | 281 | (0.62) | 0.003 | 45 | (0.79) | 291 | (0.65) | 0.017 |
| Gout | 632 | (11.03) | 4,938 | (10.96) | 0.002 | 545 | (9.51) | 3,929 | (8.72) | 0.028 |
| Obesity | 120 | (2.09) | 765 | (1.70) | 0.029 | 154 | (2.69) | 563 | (1.25) | **0.104** |
| **CVD-related comorbidities, No. (%)** | | | | | | | | | | |
| Coronary heart disease |  |  |  |  |  |  |  |  |  |  |
| Myocardial infarction | 101 | (1.76) | 901 | (2.00) | -0.017 | 125 | (2.18) | 914 | (2.03) | 0.011 |
| Cardiac arrhythmia | 292 | (5.10) | 2,366 | (5.25) | -0.007 | 320 | (5.58) | 2,320 | (5.15) | 0.019 |
| Heart failure | 143 | (2.50) | 1,221 | (2.71) | -0.013 | 174 | (3.04) | 1,328 | (2.95) | 0.005 |
| Stroke |  |  |  |  |  |  |  |  |  |  |
| Ischemic stroke | 168 | (2.93) | 1,784 | (3.96) | -0.056 | 268 | (4.68) | 2,017 | (4.48) | 0.010 |
| Hemorrhagic stroke | 59 | (1.03) | 487 | (1.08) | -0.005 | 63 | (1.10) | 523 | (1.16) | -0.006 |
| **Other comorbidities, No. (%)** | | | | | | | | | | |
| COPD | 373 | (6.51) | 2,625 | (5.82) | 0.028 | 443 | (7.73) | 2,575 | (5.71) | 0.081 |
| Depression | 269 | (4.69) | 1,777 | (3.94) | 0.037 | 290 | (5.06) | 1,821 | (4.04) | 0.049 |
| Peptic ulcer disease | 619 | (10.80) | 4,442 | (9.86) | 0.031 | 831 | (14.50) | 4,081 | (9.05) | **0.170** |
| Hyperthyroidism | 83 | (1.45) | 755 | (1.68) | -0.018 | 79 | (1.38) | 710 | (1.58) | -0.016 |
| DCSI, mean (SD) | 0.50 | (0.88) | 0.53 | (0.92) | -0.031 | 0.78 | (1.10) | 0.69 | (1.05) | 0.086 |
| CCI, mean (SD) | 1.50 | (1.91) | 1.37 | (1.94) | 0.065 | 2.12 | (2.17) | 1.78 | (2.19) | **0.158** |
| **Healthcare service utilization, No. (%)** | | | | | | | | | | |
| HbA1c test |  |  |  |  |  |  |  |  |  |  |
| 0 | 1,347 | (23.51) | 11,172 | (24.79) | 0.020 | 343 | (5.99) | 6,508 | (14.44) | **0.309** |
| 1 | 2,936 | (51.24) | 22,470 | (49.86) |  | 1,037 | (18.10) | 9,656 | (21.42) |  |
| 2 | 838 | (14.62) | 6,942 | (15.40) |  | 1,079 | (18.83) | 8,930 | (19.81) |  |
| ≥ 3 | 609 | (10.63) | 4,486 | (9.95) |  | 3,271 | (57.09) | 19,976 | (44.32) |  |
| LDL test |  |  |  |  |  |  |  |  |  |  |
| 0 | 2,883 | (50.31) | 23,364 | (51.84) | 0.034 | 1,309 | (22.84) | 14,569 | (32.33) | **0.238** |
| 1 | 1,710 | (29.84) | 13,237 | (29.37) |  | 1,387 | (24.21) | 10,849 | (24.07) |  |
| 2 | 608 | (10.61) | 4,650 | (10.32) |  | 1,111 | (19.39) | 8,459 | (18.77) |  |
| ≥ 3 | 529 | (9.23) | 3,819 | (8.47) |  | 1,923 | (33.56) | 11,193 | (24.83) |  |
| Inpatient visits |  |  |  |  |  |  |  |  |  |  |
| 0 | 4,587 | (80.05) | 35,981 | (79.83) | -0.003 | 4,590 | (80.10) | 36,880 | (81.83) | 0.045 |
| 1 | 840 | (14.66) | 6,866 | (15.23) |  | 756 | (13.19) | 5,539 | (12.29) |  |
| 2 | 203 | (3.54) | 1,488 | (3.30) |  | 207 | (3.61) | 1,500 | (3.33) |  |
| ≥ 3 | 100 | (1.75) | 735 | (1.63) |  | 177 | (3.09) | 1,151 | (2.55) |  |
| **Co-medications, No. (%)** |  |  |  |  |  |  |  |  |  |  |
| Drugs inducing steatohepatitis | | | | | | | | | | |
| Amiodarone | 26 | (0.45) | 285 | (0.63) | -0.024 | 37 | (0.65) | 349 | (0.77) | -0.015 |
| Methotrexate | 20 | (0.35) | 145 | (0.32) | 0.005 | 22 | (0.38) | 179 | (0.40) | -0.002 |
| Systemic corticosteroids | 1,628 | (28.41) | 12,100 | (26.85) | 0.035 | 1,640 | (28.62) | 11,829 | (26.25) | 0.053 |
| Valproate | 65 | (1.13) | 409 | (0.91) | 0.023 | 85 | (1.48) | 471 | (1.05) | 0.039 |
| Carbamazepine | 27 | (0.47) | 192 | (0.43) | 0.007 | 22 | (0.38) | 167 | (0.37) | 0.002 |
| Tamoxifen | 26 | (0.45) | 164 | (0.36) | 0.014 | 34 | (0.59) | 177 | (0.39) | 0.029 |
| Glucose-lowering agents |  |  |  |  |  |  |  |  |  |  |
| Metformin | 4,530 | (79.06) | 35,536 | (78.85) | 0.005 | 4,776 | (83.35) | 35,695 | (79.20) | **0.107** |
| Sulfonylureas | 1,858 | (32.43) | 15,187 | (33.70) | -0.027 | 2,627 | (45.85) | 18,929 | (42.00) | 0.078 |
| Thiazolidinediones | 90 | (1.57) | 863 | (1.91) | -0.026 | 284 | (4.96) | 2,213 | (4.91) | 0.002 |
| DPP-4 inhibitors | 374 | (6.53) | 2,779 | (6.17) | 0.015 | 1,323 | (23.09) | 8,410 | (18.66) | **0.109** |
| GLP-1RAs | NA^c^ | (NA) | NA^c^ | (NA) | 0.003 | 15 | (0.26) | 73 | (0.16) | 0.022 |
| SGLT-2 inhibitors | 4 | (0.07) | 35 | (0.08) | -0.003 | 59 | (1.03) | 440 | (0.98) | 0.005 |
| α glucosidase inhibitors | 231 | (4.03) | 2,035 | (4.52) | -0.024 | 469 | (8.18) | 3,448 | (7.65) | 0.020 |
| Glinides | 177 | (3.09) | 1,455 | (3.23) | -0.008 | 295 | (5.15) | 2,132 | (4.73) | 0.019 |
| Long-acting insulin | 107 | (1.87) | 926 | (2.05) | -0.014 | 236 | (4.12) | 1,605 | (3.56) | 0.029 |
| CVD-related medications |  |  |  |  |  |  |  |  |  |  |
| Statins | 1,982 | (34.59) | 15,760 | (34.97) | -0.008 | 2,853 | (49.79) | 22,304 | (49.49) | 0.006 |
| Fibrates | 648 | (11.31) | 4,844 | (10.75) | 0.018 | 781 | (13.63) | 5,440 | (12.07) | 0.047 |
| Ezetimibe | 106 | (1.85) | 850 | (1.89) | -0.003 | 207 | (3.61) | 1,417 | (3.14) | 0.026 |
| ACEIs | 562 | (9.81) | 5,050 | (11.20) | -0.046 | 549 | (9.58) | 4,379 | (9.72) | -0.005 |
| ARBs | 1,662 | (29.01) | 13,458 | (29.86) | -0.019 | 2,165 | (37.78) | 17,321 | (38.43) | -0.013 |
| Beta-blockers | 1,627 | (28.39) | 12,788 | (28.37) | 0.000 | 1,650 | (28.80) | 12,827 | (28.46) | 0.007 |
| CCBs | 2,228 | (38.88) | 18,191 | (40.36) | -0.030 | 2,389 | (41.69) | 19,116 | (42.41) | -0.015 |
| Loop diuretics | 575 | (10.03) | 4,692 | (10.41) | -0.012 | 599 | (10.45) | 4,738 | (10.51) | -0.002 |
| Thiazide diuretics | 677 | (11.82) | 5,538 | (12.29) | -0.015 | 722 | (12.60) | 5,793 | (12.85) | -0.008 |
| Aldosterone antagonists | 78 | (1.36) | 798 | (1.77) | -0.033 | 146 | (2.55) | 1,000 | (2.22) | 0.022 |
| Other medications |  |  |  |  |  |  |  |  |  |  |
| Aspirin (low dose) | 950 | (16.58) | 7,924 | (17.58) | -0.027 | 1,112 | (19.41) | 8,960 | (19.88) | -0.012 |
| Aspirin (high dose) | 224 | (3.91) | 1,744 | (3.87) | 0.002 | 236 | (4.12) | 1,374 | (3.05) | 0.058 |
| NSAIDs | 4,369 | (76.25) | 32,649 | (72.44) | 0.087 | 4,388 | (76.58) | 31,893 | (70.76) | **0.132** |
| Lactulose | 25 | (0.44) | 142 | (0.32) | 0.020 | 53 | (0.92) | 266 | (0.59) | 0.039 |
| Tocopherol (vitamin E) | 25 | (0.44) | 158 | (0.35) | 0.014 | 29 | (0.51) | 142 | (0.32) | 0.030 |
| DAA | 11 | (0.19) | 66 | (0.15) | 0.011 | 16 | (0.28) | 99 | (0.22) | 0.012 |
| HBV-related medications | 10 | (0.17) | 104 | (0.23) | -0.013 | 32 | (0.56) | 236 | (0.52) | 0.005 |

Abbreviations: HBV, hepatitis B virus; HCV, hepatitis C virus; CVD, cardiovascular disease; COPD, chronic obstructive pulmonary disease; CCI, Charlson Comorbidity index; DCSI, diabetes complications severity index; LDL, low-density lipoprotein; DPP-4 inhibitors, dipeptidyl peptidase-4 inhibitors; GLP-1 RAs, glucagon-like peptide-1 receptor agonists; SGLT-2 inhibitors, sodium-glucose cotransporter-2 inhibitors; ACEI, angiotensin-converting enzyme inhibitor; ARB, angiotensin II receptor blocker; CCB, calcium channel blockers; NSAID, non-steroidal anti-inflammatory drugs; DAA, direct-acting antiviral

Notes:

^a^ The index date refers to the date of first NAFLD/NASH diagnosis.

^b^ An absolute SMD of >0.1 indicates a statistically significant difference between-group difference in patient characteristics.

^c^ Use of GLP-1RAs was also measured and adjusted for in the matching procedures. However, a very low prescription of GLP-1RAs (e.g., less than 3 prescriptions) in our study period could not be shown in the table to comply with the utilization policy of Taiwan’s National Health Insurance database.

**Supplemental Figure 2. Impact of unmeasured confounders assessed using the array method (rule-in)**

**
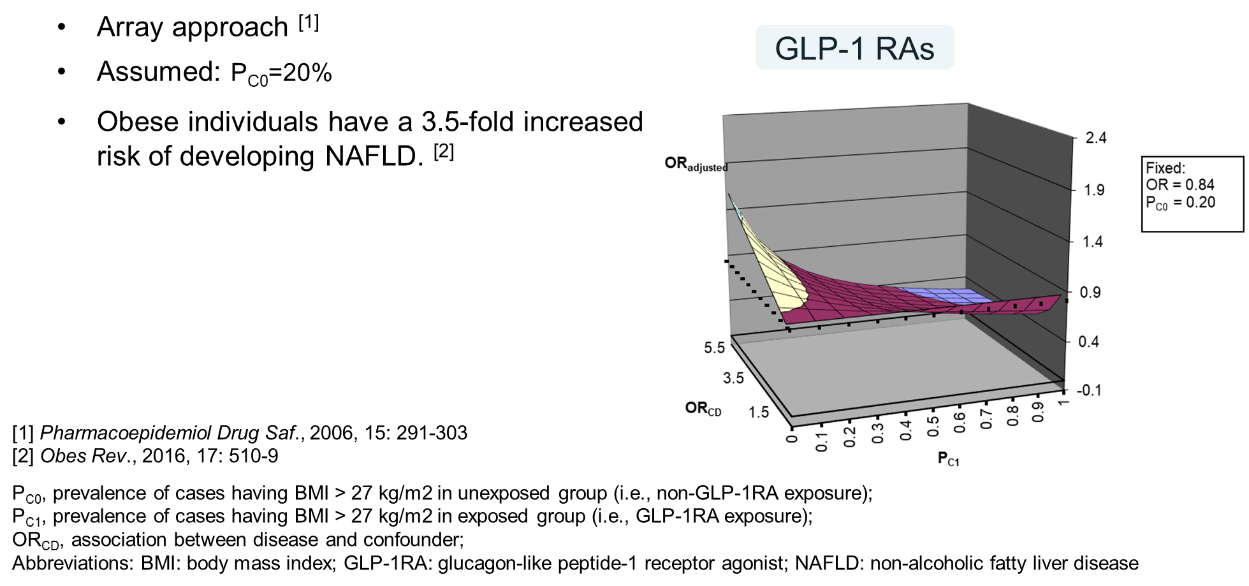
**

Length: Surface under the curve represents the “true” or fully adjusted odds ratio (OR) where an OR of non-alcoholic fatty liver diseases (NAFLDs)/non-alcoholic steatohepatitis (NASH) of 0.84 and a prevalence of body mass index (BMI) > 27 kg/m^2^ (as an unmeasured confounder of interest) (P_C0_) of 20% in the non-GLP-1RA group were assumed. According to reported data^1^, GLP-1RAs have a weight-loss effect. We thus assumed that the prevalence of BMI > 27 kg/m^2^ (P_c1_) in the GLP-1RA group was higher than 20% (P_C0_). Finally, the predicted unbiased OR (OR_adjusted_) was away from null (OR = 1).

Reference

1. Chang KC, Shao SC, Kuo S, et al. Comparative effectiveness of dulaglutide versus liraglutide in Asian type 2 diabetes patients: a multi-institutional cohort study and meta-analysis. *Cardiovasc Diabetol*. Oct 9 2020;19(1):172. doi:10.1186/s12933-020-01148-8
